# Neurons sensitive to sky compass signals in the brain of the Madeira cockroach *Rhyparobia maderae*

**DOI:** 10.1007/s00359-025-01775-0

**Published:** 2025-11-06

**Authors:** Vanessa Althaus, Naomi Takahashi, Stefanie Jahn, Jonathan Schlegel, Juliana Kolano, Erich M. Staudacher, Uwe Homberg

**Affiliations:** 1 Department of Biology, Animal Physiology, Philipps-University of Marburg, 35032 Marburg, Germany; 2 Research Center for Integrative Evolutionary Science, SOKENDAI, Shonan Village, Hayama, 240-0193 Kanagawa Japan; 3 Center for Mind Brain and Behavior (CMBB), University of Marburg and Justus Liebig University Giessen, 35032 Marburg, Germany

**Keywords:** Insect brain, Visual system, Intracellular recordings, Polarized light, Cockroach

## Abstract

Many insects are formidable navigators illustrated by homing behavior in bees and ants or regular seasonal migrations in butterflies, moths, and others. For spatial orientation, many insects rely on celestial cues, in particular the position of the sun or the polarization pattern of the blue sky generated by the sun. In all species studied celestial polarization is perceived by photoreceptors in a highly specialized dorsal rim area of the eye. Studies in various insects showed that the central complex utilizes these and other sensory inputs to create an internal compass-like representation of external space for vector navigation. Cockroaches, likewise, rely on visual and antennal input for navigational decisions mediated by the central complex. To explore the possible contribution of sky compass signals, we have characterized the responsiveness of neurons of the optic lobe and central complex of the Madeira cockroach *Rhyparobia maderae* to the angle of polarized light and the azimuth of unpolarized light spots representing the sun or the chromatic gradient of the sky. Strong responses to polarization angle and to changing polarization angle were found in several cell types connecting both optic lobes. Responses to sky compass signals in neurons of the central complex were less pronounced, but were significant in several cell types corresponding to neurons encoding sun compass signals in other species. Although the Madeira cockroach is a nocturnal scavenger and the existence of a specialized dorsal eye region has not been established, sky compass signals likely play a substantial role in behavioral decisions.

## Introduction

**Fig. 1:**
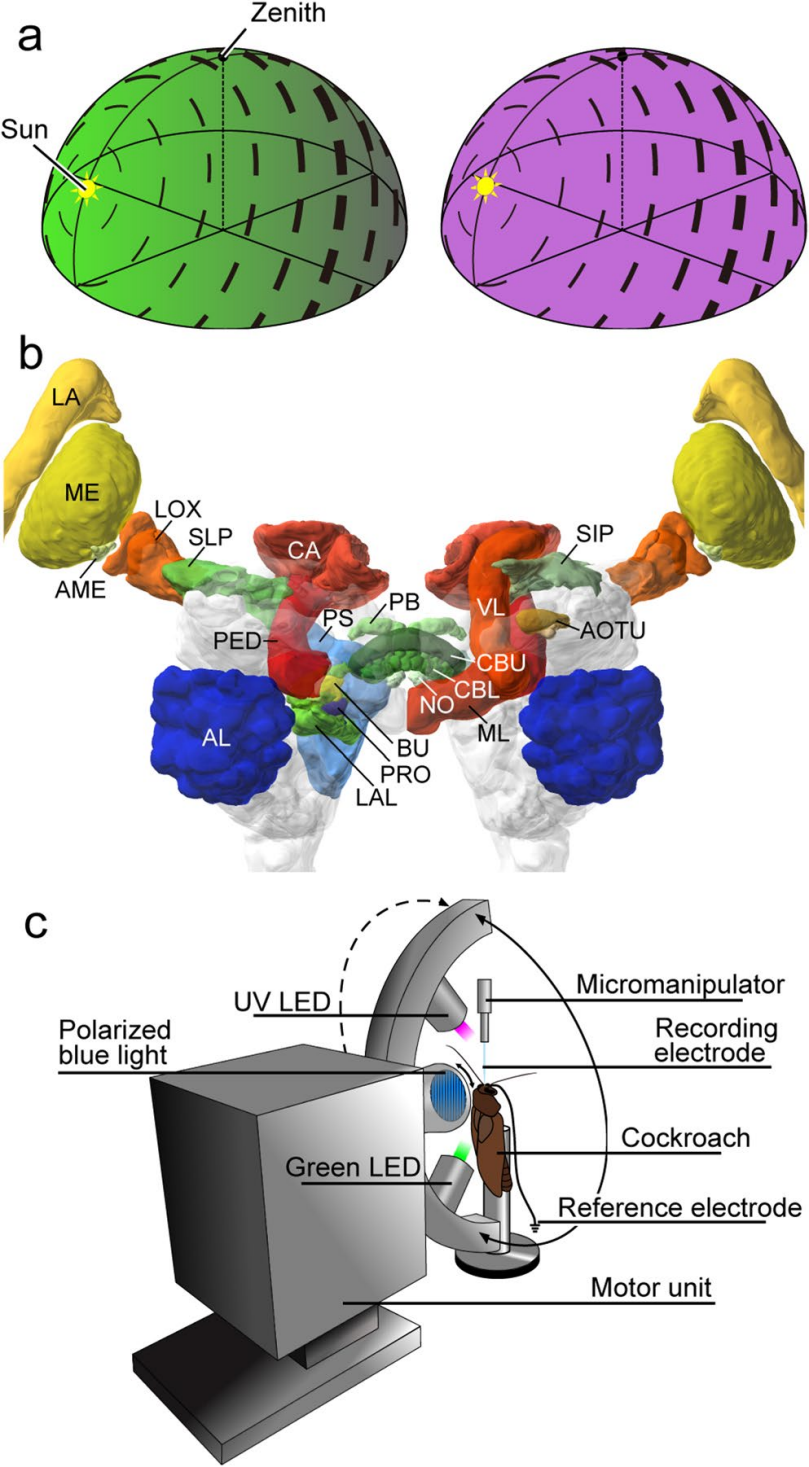
Celestial compass cues, cockroach brain organization, and recording setup. **a** Schematic illustration of the polarization pattern and color gradient in the sky. The angles of polarization (black bars) are oriented tangentially along concentric circles around the sun. The degree of polarization illustrated by the thickness of the bars increases from direct sunlight (unpolarized) to a maximum of around 70% along a great circle 90° away from the sun under ideal conditions. The spectral distribution across the sky is characterized by a gradient of long-wavelength light (left, green) and uniform distribution of short-wavelength light (right, UV) across the sky. Modified from Takahashi et al. (2022). **b** Three-dimensional frontal reconstruction of the brain of the Madeira cockroach with relevant neuropils illustrated in colour. Image obtained from https://insectbraindb.org/ (Heinze et al. 2021) based on Althaus et al. (2022). AL, antennal lobe; AME, accessory medulla; AOTU, anterior optic tubercle; CA, calyx of the mushroom body; BU, bulb; CBL, lower division of the central body; CBU, upper division of the central body; LA, lamina; LAL, lateral accessory lobe; LOX, lobula complex; ML, medial lobe; NO, noduli; PED, pedunculus; PRO, prong; PS; posterior slope; SIP, superior intermediate protocerebrum; SLP, superior lateral protocerebrum; VL, vertical lobe. **c** Recording setup. The cockroach is mounted with its head uppermost to a metal holder. Blue light, provided from the zenith, is polarized by a linear polarization filter which can be rotated 360° in clockwise or counterclockwise direction. A green and a UV LED are mounted on a rotatable arm at an elevation of 45° relative to the head of the cockroach and can be moved 360° around the cockroach head. The position anterior to the cockroach, here the position of the UV light, is defined as 0°. The recording electrode was advanced into the cockroach brain from frontal direction.

Orientation in space is a fundamental requirement of freely moving animals. It allows finding food and mating partners, returning to places of interest (feeding place, nest), and performing seasonal migrations to avoid unfavorable living conditions. In order to execute these behaviors, animals monitor the spatial relations between different places through estimates of distances, directions, and relative positions. Spatial directions are often determined in relation to landmarks, as illustrated by the coding properties of mammalian head direction cells (Taube 2007) or in relation to external compass signals provided by the sun, the stars, or the earth magnetic field as shown in many seasonal migrants (Wehner 1984; Gould 1998; Frost and Mouritsen 2006). Insects often rely on celestial compass information for estimating spatial directions (Wehner 1984; Merlin et al. 2012). In addition to be guided directly by the sun as the brightest spot in the sky, many insect species, notably honeybees, ants, monarch butterflies, and dung beetles, can also exploit the chromatic gradient and the polarization pattern of the sky, both of which depend on the sun’s position (Fig. 1a).

An analysis of polarization vision and its neural basis in insects has led to the identification of specific brain areas involved in sky compass navigation (Homberg et al. 2011, 2023; Hardcastle et al. 2021; Heinze 2014). In many insect species, a narrow dorsal rim area of the compound eye is modified for high polarization sensitivity. Specific adaptations include homochromacy of photoreceptors, precise alignment of rhabdomeric microvilli, and large acceptance angles for high absolute sensitivity (Labhart and Mayer 1999). The central processing of signals from the dorsal rim area has been studied in the monarch butterfly (Heinze and Reppert 2011; Nguyen et al. 2021, 2022), the field cricket (Sakura et al. 2008), dung beetles (el Jundi et al. 2015), bees (Pfeiffer and Kinoshita 2012; Stone et al. 2017), the fruit fly (Hardcastle et al. 2021; Garner et al. 2024), and the desert locust (Vitzthum et al. 2002; Heinze and Homberg 2009; Bockhorst and Homberg 2015; Pegel et al. 2018, 2019; Zittrell et al. 2020; Takahashi et al. 2022; Hensgen et al. 2022; Homberg et al. 2023). Neurons sensitive to polarization angle were characterized in the optic lobe (Labhart 1988; Labhart and Petzold 1993; el Jundi et al. 2011; Hardcastle et al. 2021), the anterior optic tubercle (Pfeiffer et al. 2005; el Jundi and Homberg 2012; Hardcastle et al. 2021), and, most intensely, in the central complex (CX), a group of neuropils extending across the brain midline, which plays a fundamental role as an internal compass for spatial orientation and goal directed motor control (Heinze and Homberg 2007; Pfeiffer and Homberg 2014; Honkanen et al. 2019; Hardcastle et al. 2021; Wilson 2023; Heinze 2023).

The Madeira cockroach *Rhyparobia maderae* is a nocturnal gregarious insect with circumtropical distribution (Cornwell 1968) and a model system for studies on circadian rhythms (Homberg et al. 2003; Stengl et al. 2015). The master circadian pacemaker in the accessory medulla of the cockroach brain has been investigated in great depth, in particular with respect to the generation of rhythmic activity (Werckenthin et al. 2012, 2020; Stengl et al. 2015), light entrainment (Schendzilorz and Stengl 2014; Arnold et al. 2019), output and circadian coupling pathways (Reischig et al. 2004; Wei et al. 2014). Loesel and Homberg (2001) showed that two commissural neurons, connecting the medulla and accessory medulla of both optic lobes, are highly sensitive to the angle of polarization (AoP) of polarized light. This raises the possibility that *R. maderae*, like other species, uses sky compass signals for spatial orientation. To further explore this hypothesis, we have analyzed the anatomical organization of the *R. maderae* brain (Fig. 1b; Rosner et al. 2017; Althaus et al. 2022) with particular focus on the CX (Jahn et al. 2023, 2024). The data show that components of the pathway providing sky compass signals to the CX in bees, flies, and locusts — particularly the connection from the anterior optic tubercle to the central body — are also present in the cockroach brain, although the volume of the anterior optic tubercle is highly reduced (Althaus et al. 2022). The CX of the cockroach exhibits some specific features, especially in the organization of the input stage to the sky compass network, compared to other insects (Jahn et al. 2023, 2024). Evidence from another blaberid species, the discoid cockroach *Blaberus discoidalis*, nevertheless, demonstrated a role of the CX in spatial visual orientation and motor control (Ritzmann et al. 2012; Varga et al. 2017), but the significance of sky compass signals has not been tested. To expand the range of insects analyzed for sky compass coding to include a nocturnal scavenger, we used intracellular recordings to investigate the sensitivity of neurons of the optic lobe and CX of the Madeira cockroach to sky compass cues, including polarization angle and simulated solar azimuth.

## Materials and methods

### Animals

Cockroaches (*Rhyparobia maderae*, previously termed *Leucophaea maderae*) were raised in crowded colonies at the Department of Biology, University of Marburg. Animals were kept in boxes at a temperature of around 25 °C and were reared under a 12:12 h day/night rhythm. Adults of both sexes, mainly females, were used for the experiments. All animal procedures were performed according to the guidelines of the European Union (Directive 2010/63/EU) and the German Animal Welfare Act.

## Preparation

Animals were taken from the colony at the end of the light phase. They were anesthetized either by CO_2_ and iced water for at least 1 h or were cold anesthetized overnight at 4 °C. The next day they were fixed with dental wax to a special metal holder with their heads uppermost. The cockroach head was oriented with its frontal face horizontally and was again fixed with dental wax to the holder. The head capsule was opened frontally between both compound eyes using a broken razor blade. A small rectangular piece of cuticle was removed without injuring the bases of the antennae, the eyes, and the mouthparts. Trachea and fat tissue above the brain was removed with fine forceps, while the brain was kept moist with cockroach saline (4.088 g NaCl, 0.186 g KCl, 0.102 g MgCl_2_ × 6 H_2_O, 0.168 g NaHCO_3_, 0.4125 g NaH_2_PO_4_, 0.801 g glucose, dissolved in 0.5 l H_2_O, pH 7.25). The esophagus was cut and removed through the abdomen to reduce movements. For stabilizing the brain, a twisted wire, formed as a spoon, was positioned below the brain. Finally, the neural sheath covering the brain was partly removed with fine forceps.

## Intracellular recording and stimulation

 Intracellular recordings were performed inside a Faraday cage covered with dark cloth on all sides except the front. Computer screens were covered with red Perspex sheets, resulting in diffuse dim red background light when no stimulus was presented. We used sharp glass electrodes (resistance 70–220 MΩ) drawn from borosilicate glass capillaries (Hilgenberg, Malsfeld, Germany) with a Flaming/Brown horizontal puller (P-97, Sutter Instrument, Novato, CA, USA) for recording neural activity. The electrode tips were filled with 4% Neurobiotin (Vector Laboratories, Burlingame, CA, USA) dissolved in 1 mol l^−1^ KCl and the shanks backed up with 1 mol l^−1^ KCl. A chlorinated silver wire inserted into the open head capsule served as the reference electrode. The recorded signals were amplified 10x with an amplifier (BA-01x or SEC 05 L, npi Electronic Instruments, Tamm, Germany), monitored by an audio monitor, and visualized with an oscilloscope. Data were sampled at 20 kHz using a digital interface (Power1401-mkII, Cambridge Electronic Design, Cambridge, UK) and stored on a PC using Spike2 (version 7.06 and 7.14, Cambridge Electronic Design, Cambridge, UK). After recording a neuron of interest, Neurobiotin solution was injected by applying a positive current (0.3–1.5 nA) for 1–5 min.

Neuronal activity was recorded during the presentation of different visual stimuli that contribute to sky compass coding (Fig. 1c). The cockroach was positioned with its dorsal side to the light stimuli (Fig. 1c). Blue light, generated by a light emitting diode (LED, 447.5 nm, LXML-PR01-0500, Philips Lumileds, intensity 1.7 × 10^13^ photons cm^−2^ s^−1^), was linearly polarized by a polarization filter (polarizer, HNP’B, Polaroid) in front of the LED. The stimulus was positioned dorsal to the cockroach head (Fig. 1c) and covered a visual angle of 32.5°. The polarizer was rotated through 360° at an angular velocity of 30°, 40°, or 60° s^−1^ in clockwise (CW, right turn, 0°−360°) and counterclockwise (CCW, left turn, 360°−0°) direction. Rotation started at an *E*-vector orientation of the polarizer parallel to the cockroach’s longitudinal body axis, defined as 0°. In addition, the cockroach was stimulated with an unpolarized green (LED, 527 nm, Oslon SSL 80 LT CP7P, Osram Opto Semiconductors, intensity 10^14^ photons cm^−2^ s^−1^) and ultraviolet (UV; LED, 365 nm, NCSU033B(T), Nichia, intensity 10^14^ photons cm^−2^ s^−1^) light spot to represent unpolarized sunlight or the chromatic contrast across the sky (Fig. 1). The intensities of these stimuli were lower than direct sunlight (up to 12 × 10^16^ photons cm^−2^ s^−1^) but higher than direct moonlight (up to 3 × 10^11^ photons cm^−2^ s^−1^; Björn and Vogelmann 1994). The light spots (visual angle 16.3°) were moved at an elevation of 45° and an angular velocity of 40° or 60° s^−1^ around the head of the cockroach along a course indicated by the solid and dashed circle in Fig. 1c. The position of the light spots anterior to the cockroach was defined as 0°. If possible, three consecutive full rotations of the three stimuli were presented. The first rotation was excluded from further evaluation, because onset of the different stimuli often produced a phasic response which should not affect the statistical test.

## Histology and image acquisition

After recording and Neurobiotin injection, brains were dissected and fixed overnight at 4 °C in a solution consisting of 4% paraformaldehyde, 0.25% glutaraldehyde, 0.2% saturated picric acid in 0.1 mol l^−1^ phosphate buffered saline (PBS, 0.15 mol l^−1^ NaCl in 0.1 mol l^−1^ sodium phosphate buffer, pH 7.4). After fixation, the brains were rinsed 4 × 15 min in 0.1 mol l^−1^ PBS, or stored in sodium phosphate buffer at 4 °C until further processing. For visualizing the Neurobiotin tracer, the brains were incubated in Cy3-conjugated streptavidin (1:1000, Dianova, Hamburg, Germany) in PBT (0.1 mol l^−1^ PBS containing 0.3% Triton X-100) for 3 days at 4 °C in the refrigerator. Afterwards the brains were washed 2 × 30 min in PBT, rinsed 3 × 20 min in PBS, and dehydrated by an ascending ethanol series (30%, 50%, 70%, 90%, 95% and 2 × 100% ethanol, 15 min each). The brains were cleared by incubation in a 1:1 ethanol/methyl salicylate solution for 20 min followed by 100% methyl salicylate for at least 30 min. Finally, the brains were mounted in Permount (Fisher Scientific, Pittsburgh, PA) between two coverslips.

The whole mount preparations were scanned by a confocal laser scanning microscope (Leica, TCS SP5, Leica Microsystems, Wetzlar, Germany) with a 20x oil immersion objective (HC PL APO 20x/0.75 lmm Corr CS2). Images were acquired at a resolution of 1024 × 1024 pixels in the xy-plane (pixel size 0.75 μm x 0.75 μm) and a step size of 1–2 μm in z direction. Scanning frequency was 400 Hz. Cy3 fluorescence was excited by a diode-pumped solid-state laser (561 nm, DPSS 10 mW). Image stacks were merged with the software Amira 6.5 (ThermoFisher Scientific, Waltham, MA, USA). Images were processed and figures created using Adobe Photoshop and Adobe Illustrator 2021 (version 25.2.1, Adobe Systems, San José, CA, USA) and Affinity Photo (Serif Inc., Nottingham, UK). The nomenclature for brain areas follows Althaus et al. (2022), for neuronal cell types of the optic lobe Loesel and Homberg (2001), and for neurons of the CX Jahn et al. (2023, 2024). Nomenclature for CX neuron types in the fruit fly *Drosophila melanogaster* follows Hulse et al. (2021).

## Physiological data analysis and plots

Spike time-stamps were extracted by threshold-based event detection using Spike2. Mean spiking frequencies were calculated and displayed using the Spike2 implementation of a moving average (window size 1 s). Background activity (mean ± SD) was calculated from 1-s bins of parts of spike train segments in which the stimuli were turned off. Data were exported from Spike2 as *.mat-files and further analyzed in MATLAB (MathWorks, Natick, MA, USA) using the CircStat toolbox for MATLAB (Berens 2009). Time-stamps for each action potential (AP) occurring during a stimulus period were converted to angular values (0°–359.999°) separately for each repeat (trial). Circular histograms were generated with the CircHist package in MATLAB (Zittrell 2020) as described in Zittrell et al. (2020). Spikes were pooled and counted within 10, 15, or 20 deg bins (firing rate). Mean responses (± SD) per bin were calculated by averaging across all clockwise/counterclockwise trial pairs. The number of samples used in all statistical descriptions and tests corresponds to the number of bins. In polar histograms, they are shown as colored bars and black T-lines, respectively.

We calculated descriptive statistical values such as the stimulus angle eliciting maximal excitation (Φ_max_), its standard deviation, its 95% confidence interval, and the length *r* of the mean vector. For stimulation with the green and UV light spots, Φ_max_ was calculated according to the method for unimodal samples (Batschelet 1981). The rotating polarizer has a periodicity of 180°. To account for the bimodal nature of data during 360° polarizer rotation, the correlated angles were doubled to allow the use of circular statistics (Zar 1999). The result was then divided by two, yielding the Φ_max_ angle. The tuning angle (Φ_max_) in the polar histograms is indicated by an orange line, its black portion represents the length *r* of the mean vector, quantifying vector strength. This value serves as a measure of response amplitude (Bockhorst and Homberg 2015) and ranges from zero to unity. Correlation of neuronal firing rates with stimulus angles was tested by linear-circular correlation analysis employing the *circ-corrcl* function from the CircStat toolbox (Berens 2009). The coefficient of determination (*R*^2^, range 0‒1) indicates the proportion of variability in spiking rate explained by the stimulus (AoP, green, or UV azimuth; Pegel et al. 2019). *R*^2^ values are only provided in case of significant correlations (*p* < 0.05).

## Results

This study is based on 33 intracellular recordings from neurons in the cockroach (*R. maderae*) brain combined with Neurobiotin injections for cell type identification. Eight recordings were from neurons with ramifications in the optic lobe and 25 recordings from neurons innervating parts of the CX. Most neurons were stimulated with polarized light and a fraction of those, with the unpolarized UV and green light spots (Table 1).

**Table 1:** Sensitivity to polarization angle of blue light and azimuth of the green and UV light

Cell type	Polarized light				Green light				UV light				Figures
	*n* ^a^	Φ_max_	*r*	*R* ^*2*^	*n*	Φ_max_	*r*	*R* ^*2*^	*n*	Φ_max_	*r*	*R* ^*2*^	
LOX-PN1	8	128.2°	0.07	0.53	n.t.^b^				n.t.				Figure 2
OC2	4	n.s.^c^			4	177.0°	0.25	0.47	4	195.6°	0.36	0.54	Figure 3
PC2	8	35.6°	0.24	0.16	n.t.				n.t.				Figure 4a–d
PC3	6	136.4°	0.16	0.14	n.t.				n.t.				Figure 4e–h
PC4	2	51.8°	0.67	0.86	n.t.				n.t.				Figure 5a–c
PC5	4	45.7°	0.13	0.72	4	84.4°	0.08	0.57	4	94.3°	0.59	0.95	Figure 5d–f
SOC1	6	n.s.			n.t.				n.t.				Figure 6a–e
SOC2	6	24.5°	0.21	0.11	n.t.				n.t.				Figure 6f–h
TL2,3b-TH	4	122.6°	0.28	0.19	n.t.				n.t.				Figure 7a–d
TL	2	n.s.			n.t.				n.t.				
TB1d	2	113.0°	0.09	0.79	2	28.2°	0.03	0.31	2	293.7°	0.04	0.29	Figure 7e–g
TB1c	8	n.s.			n.t.				n.t.				Figure 7h, i
TB4	6	n.s.			4	n.s.			4	n.s.			
CU2c	8	n.s.			n.t.				n.t.				
CU2d	8	n.s.			n.t.				n.t.				
CP2	4	n.s.			n.t.				n.t.				
CPU1a-1	6	124.8°	0.04	0.37	n.t.				n.t.				Figure 8a–c
CPU1a-3	4	15.3°	0.14	0.21	n.t.				n.t.				Figure 8d–h
CPU1a	2	n.s.			2	n.s.			2	n.s.			
CPU5	4	n.s.			n.t.				n.t.				
TU_VES_1	4	n.s.			n.t.				n.t.				
TU_VES_4b	4	n.s.			n.t.				4	n.s.			
TU_VES_4b	2	n.s.			2	n.s.			2	n.s.			Figure 9a, b
TU_VES_4c	4	n.s.			n.t.				n.t.				
TU_VES_3	6	n.s.			n.t.				n.t.				
TU_PS_3	4	n.s.			n.t.				n.t.				
POU2a	6	n.s.			2	n.s.			2	n.s.			
POU2a	2	n.s.			2	n.s.			2	n.s.			
POU1	2	n.s.			4	224.0°	0.02	0.19	4	n.s.			Figure 9e, f
POU2b	4	n.s.			4	n.s.			4	n.s.			
POU1	4	n.s.			2	n.s.			2	n.s.			
POU1	2	n.s.			n.t.				n.t.				
POU3b	4	174.9°	0.03	0.18	n.t.				n.t.				Figure 9c, d

^a^*n* = total number of rotations, consisting of equal numbers of clockwise and counterclockwise rotations

^b^n.t. = not tested

^c^n.s. = not significant

## Neurons of the optic lobe

We obtained eight recordings from neurons with ramifications in the optic lobe, including one recording from a projection neuron of the lobula complex (LOX), one recording from a centrifugal neuron innervating the LOX and medulla, and six recordings from commissural neurons connecting the optic lobes of both hemispheres. Of these, seven neurons were sensitive to polarization angle. Two neurons were tested for responses to the UV and green light spots, and both showed azimuth dependent responses to both stimuli (Table 1).

## Neurons with unilateral optic lobe innervation

**Fig. 2:**
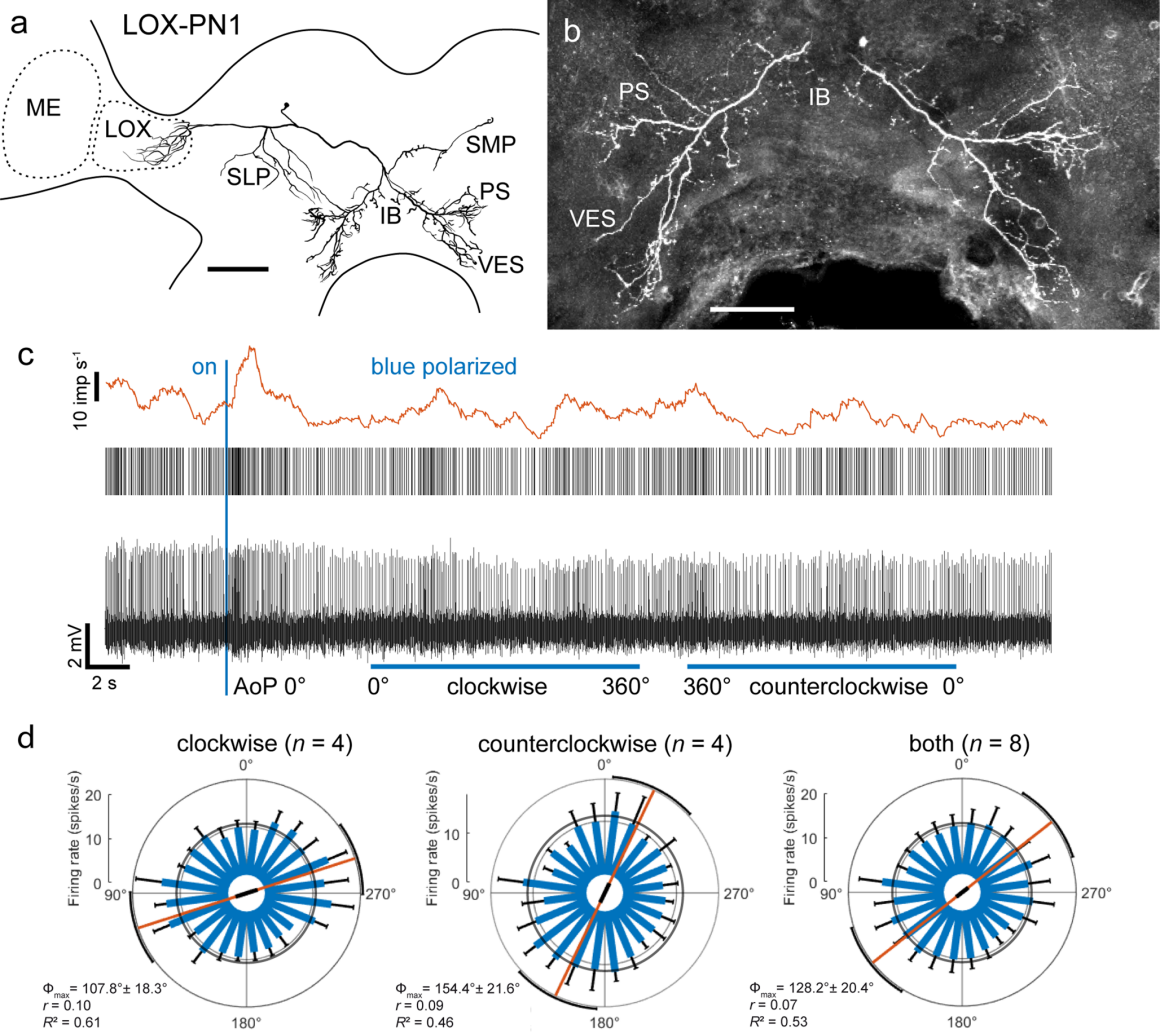
Morphology and physiology of lobula projection neuron LOX-PN1. **a** Frontal reconstruction of LOX-PN1 with fine arborizations in the anterior lobe of the lobula complex (LOX) and the superior lateral protocerebrum (SLP). The axonal fiber gives rise to varicose ramifications in the contralateral superior medial protocerebrum (SMP) and, bilaterally symmetric arborizations in the posterior slope (PS), vest (VES), and inferior bridge (IB). **b** Stack of confocal images illustrating varicose ramifications of LOX-PN1 in the IB, PS and VES. **c** Intracellular recording (bottom trace), raster plot (middle trace), and mean spike frequency illustrating neural activity in the dark, after turning on blue polarized light (blue vertical bar) and during a clockwise and counterclockwise rotation of the polarizer (blue horizontal bars; angular velocity 30° s^−1^). **d** Circular diagrams showing mean spiking activities during clockwise (left), counterclockwise (middle), and all rotations (left) of the polarizer. Spiking activity (blue bars) is plotted in 15° bins, black T-bars indicate SD, and grey circles show background spiking activity. The neuron responded significantly to the rotation of the polarizer (blue light) during all rotations. Orange lines with black bars (length of mean vector *r*) indicate the angle of polarization (AoP) eliciting maximal excitation (Φ_max_), and its 95% confidence interval (black arcs). Scale bar = 200 μm (a), 100 μm (b)

The projection neuron of the LOX, termed LOX-PN1, had its soma in the anterior dorsal soma rind of the brain above the crepine (Fig. 2a). The neuron had fine arborizations in the anterior lobe of the LOX, the posterior superior lateral protocerebrum and the posterior slope (Fig. 2a). The main neurite projected to the brain midline and sent bilaterally symmetric varicose processes into the posterior slope, vest, and inferior bridge (Fig. 2b) and, in the contralateral hemisphere, to the superior medial protocerebrum. The neuron had a background activity of 13.7 ± 2.1 (SD, *n* = 6) impulses (imp) s^−1^ and was phasically excited when turning the blue polarized light (AoP 0°) on and off (Fig. 2c). The neuron was significantly tuned to the AoP. It showed slight differences in the polarization angle eliciting maximal excitation with Φ_max_ at 107.8° for clockwise and 154.4° for counterclockwise rotation of the polarizer (Fig. 2d). Similar differences have commonly been observed in polarization-sensitive neurons of other species (e.g. Bockhorst et al. 2015). For a comparison with other neurons, evaluation of all rotations (4 clockwise and 4 counterclockwise) revealed a preferred AoP at Φ_max_ = 128.2°. Responses to green and UV light spots were not tested.

**Fig. 3:**
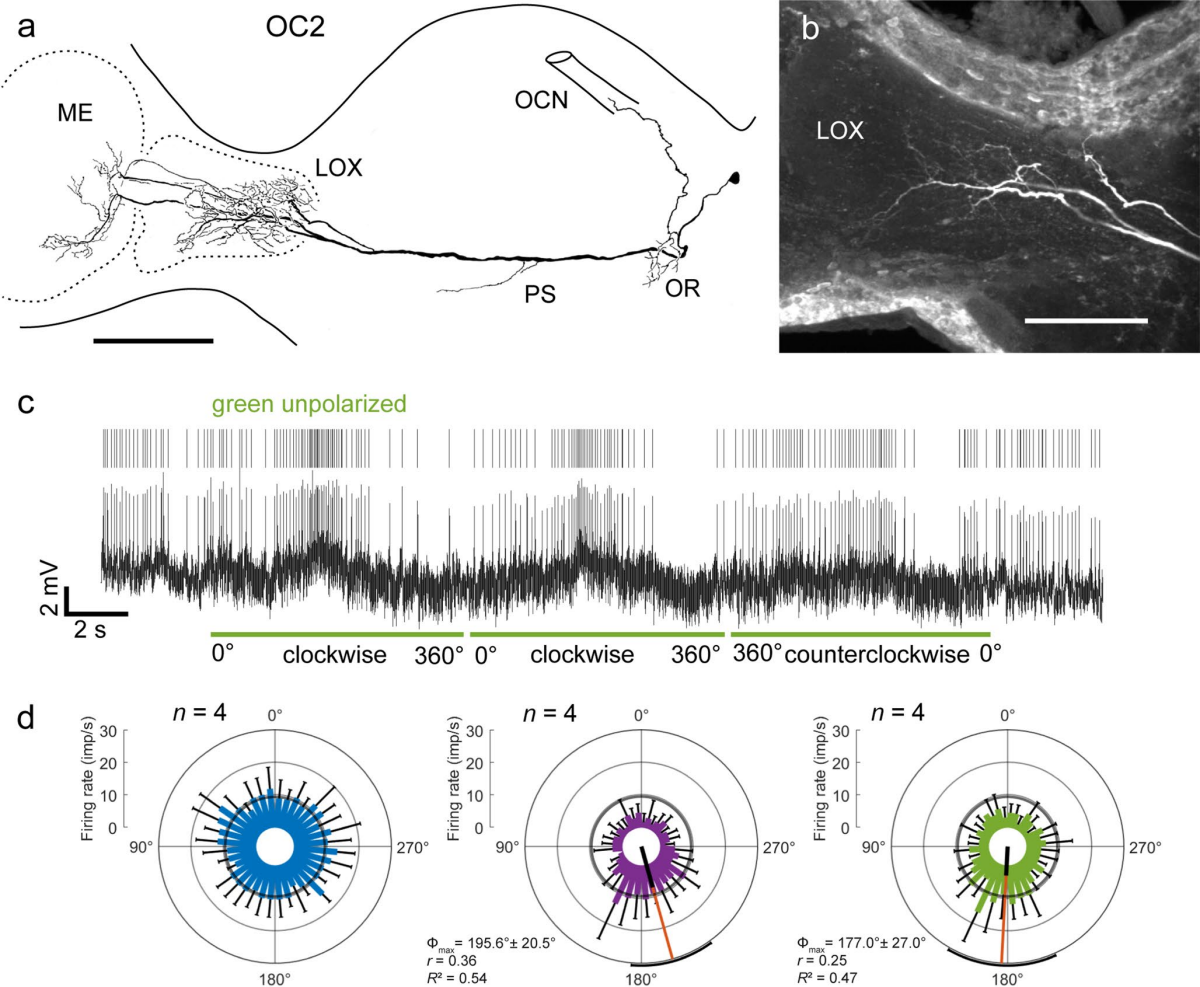
Ocellar interneuron OC2. **a** Frontal reconstruction of the neuron. The neuron has a single fiber in the ocellar nerve (OCN) and proximal arborizations in the ocellar root (OR) and the posterior slope (PS). A centrifugal neurite extends to the optic lobe and gives rise to beaded arborizations in the lobula complex (LOX) and a proximal layer of the medulla (ME). **b** Stack of confocal images illustrating ramifications of OC2 in the LOX. **c** Intracellular recording (bottom trace) and raster plot (top trace) illustrating changes in spiking during clockwise and counterclockwise movement of the green unpolarized LED around the head of the cockroach (angular velocity 40° s^−1^). Before, between, and after rotations the animal was exposed to the stationary LED in front of the animal (0° azimuth). **d** Circular plots of spiking activity of the OC2 neuron from four (*n*) rotations (2 clockwise, 2 counterclockwise) of the polarizer with polarized blue light (left), an unpolarized UV light spot (middle), and a green light spot (right). Spiking activity is plotted in 10° bins, black T-bars indicate SD, grey circles indicate background activity. The neuron is not sensitive to AoP but shows a preferred azimuth, illustrated by an orange line with black bar (length of mean vector *r*), and a black arc (95% confidence interval) for the unpolarized UV and green light spot. Responses to the unpolarized lights show spatial opponency with strong inhibition at stimulus azimuths opposite from Φ_max_. Scale bars = 200 μm (a), 100 μm (b)

The centrifugal neuron of the optic lobe (Fig. 3) can be regarded as a second-order ocellar neuron. It was termed OC2, as it is the second ocellar interneuron identified in *R. maderae* innervating the optic lobe (Loesel and Homberg 2001). The soma of OC2 was located in the pars intercerebralis near the midline, above the protocerebral bridge (PB). The neuron had fine ramifications in the ipsilateral cerebrum concentrated in the ocellar root, lateral parts of the inferior bridge and, most extensively, in parts of the posterior slope (Fig. 3a). One fiber could be traced into the ipsilateral ocellar tract and nerve (Fig. 3a). The main neurite extended to the optic lobe and gave rise to beaded arborizations in the anterior lobe of the LOX and a proximal layer of the medulla (Fig. 3a, b). The neuron had a background activity of 9.4 ± 1.7 (SD, *n* = 7) imp s^−1^ and was insensitive to the AoP (Fig. 3d). In contrast, the neuron was highly sensitive to the position of the UV (Φ_max_ = 195.6°) and green light stimulus (Φ_max_ = 177.0°). The neuron showed spatial opponency to both light spots (Fig. 3c, d) with strong inhibition at angles opposite to Φ_max_ (Φ_min_). The neuron was phasically excited by turning the light stimuli on (all wavelengths), but showed no response at lights off (not shown).

### Neurons connecting both optic lobes

**Fig. 4:**
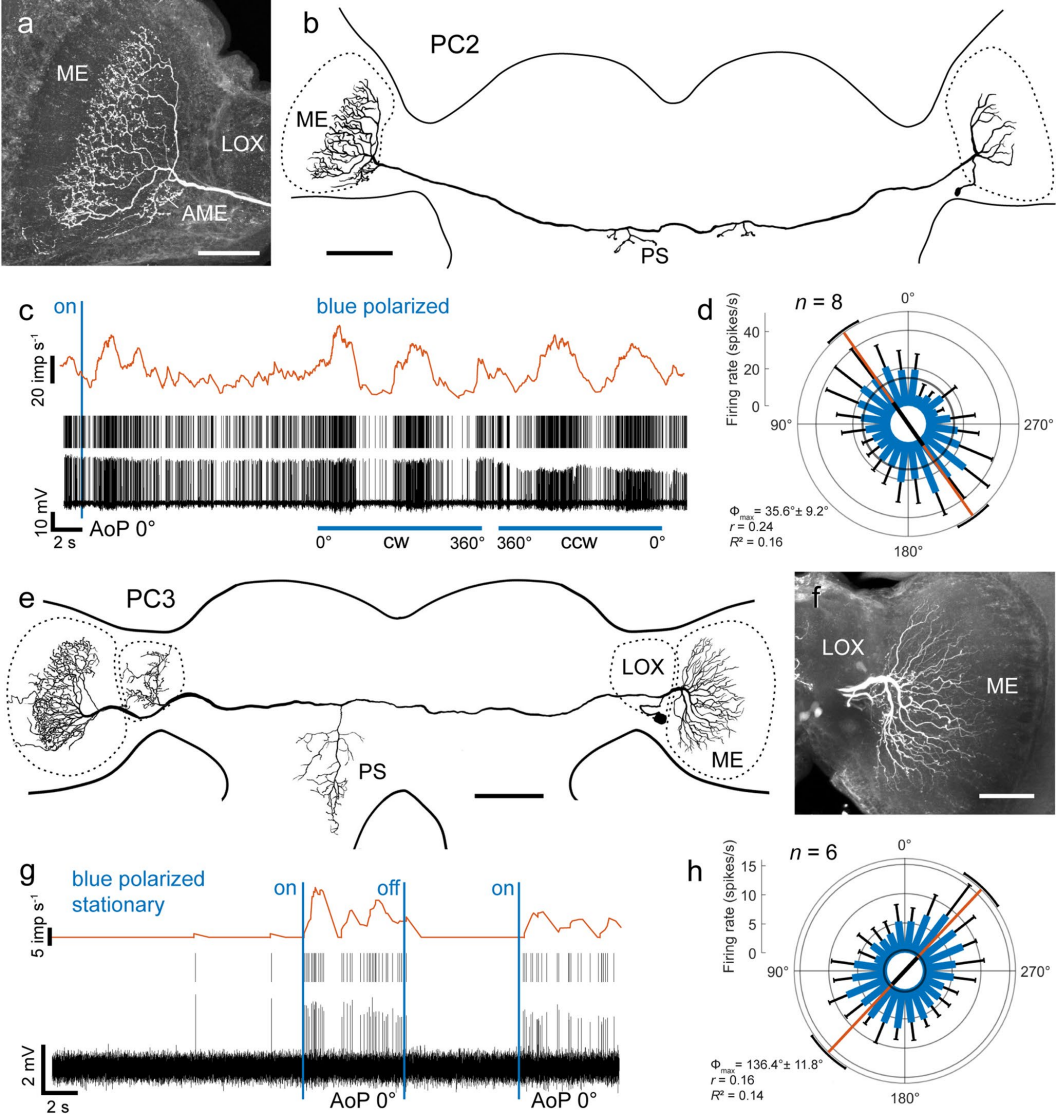
Commissural neurons PC2 and PC3 connecting both optic lobes. **a** Stack of confocal images of PC2 in the left optic lobe and **b** frontal reconstruction of the recorded PC2 neuron. It innervates both medullas (ME), the contralateral accessory medulla, and has bilateral short beaded side branches in the posterior slope (PS). **c** Intracellular recording (bottom trace), raster plot (middle trace), and mean spike frequency illustrating neural activity in the dark, after turning on blue polarized light (blue vertical bar, AoP 0°), and during a clockwise (cw) and counterclockwise (ccw) rotation of the polarizer (blue horizontal bars; angular velocity 30° s^−1^). **d** Circular plot of spiking activity of the PC2 neuron from eight (n) rotations (4 clockwise, 4 counterclockwise) of the polarizer. Spiking activity is plotted in 15° bins (blue bars), black T-bars indicate SD, grey circle indicates background activity. The neuron shows polarization opponency with a preferred AoP at Φ_max_ = 35.6°, illustrated by the orange line with black bar (length of mean vector *r*), and black arcs (95% confidence interval). **e** Frontal reconstruction of a PC3 neuron. The neuron arborizes in both medullas (ME) and the contralateral lobula complex (LOX). Further arborizations are in the contralateral posterior slope (PS). **f** Stack of confocal images showing arborizations of the neuron in the right optic lobe. **g** Intracellular recording (bottom trace), raster plot (middle trace), and mean spike frequency illustrating neural activity in the dark, and after turning blue polarized light (blue vertical bars, AoP 0°) on and off. **h** Circular plot of spiking activity of the PC3 neuron from six rotations (3 clockwise, 3 counterclockwise; angular velocity 30° s^−1^) of the polarizer. Spiking activity is plotted in 15° bins (blue bars), black T-bars indicate SD, grey circle indicates background activity in the dark. The neuron is excited by all AoPs with a preferred AoP at Φ_max_ = 136.4°, illustrated by the orange line with black bar (length of mean vector *r*) and black arcs (95% confidence interval). Scale bars = 200 μm (b, e), 100 μm (a, f)

Two of the six commissural cell types connecting both optic lobes, termed PC2 and PC3, had been characterized before by Loesel and Homberg (2001). As reported by Loesel and Homberg (2001), the PC2 neuron recorded here had its soma near the accessory medulla. The neuron connected medial layers of the medulla on both sides of the brain and had small side branches in the contralateral accessory medulla (Fig. 4a, b). Arborizations in the contralateral optic lobe had a varicose appearance. The axonal fiber crossed the brain midline in the posterior optic commissure and sent off small beaded processes bilaterally to the midline into the posterior slope anterior to the posterior optic tubercles. The neuron had a background activity of 15.2 ± 5.38 (SD, *n* = 6) imp s^−1^, was briefly inhibited followed by a more prominent increase in spiking activity when blue polarized light was turned on (AoP 0°), and showed polarization opponency during rotation of the zenithal polarizer (Fig. 4c, d). The preferred AoP determined from four clockwise and four counterclockwise rotations was at Φ_max_ = 35.6° (clockwise 32.3°, counterclockwise 37.7°, not shown), similar to the tuning of one PC2 neuron (Φ_max_ = 38°) studied by Loesel and Homberg (2001). Stimulation with unpolarized UV or green light was not tested.

The PC3 neuron had extensive ramifications in median layers of both medullas and in the contralateral LOX, confined to the anterior lobe (Fig. 4e). Arborizations in the ipsilateral medulla were fine (Fig. 4f), those in the contralateral optic lobe beaded. The soma of PC3 was located posteriorly below the LOX. The commissural neurite crossed the brain midline in the posterior optic commissure. Further arborizations were located in the contralateral posterior slope. The neuron had very low background spiking activity at 0.35 ± 0.65 (SD, *n* = 23) imp s^−1^ in darkness, but was strongly excited when the light was turned on (Fig. 4g). The neuron was significantly sensitive to AoP with maximum activity between 120° and 150° (clockwise rotations: Φ_max_ = 123.6°; counterclockwise rotations: Φ_max_ = 149.1°; all rotations: Φ_max_ = 136.4°). Unlike reported by Loesel and Homberg (2001) no bursting activity during light stimulation was observed, and the preferred AoP, reported by Loesel and Homberg (2001) in two recordings at Φ_max_ = 72° and 77°, likewise, differed. Stimulation with unpolarized UV or green light was not accomplished.

**Fig. 5:**
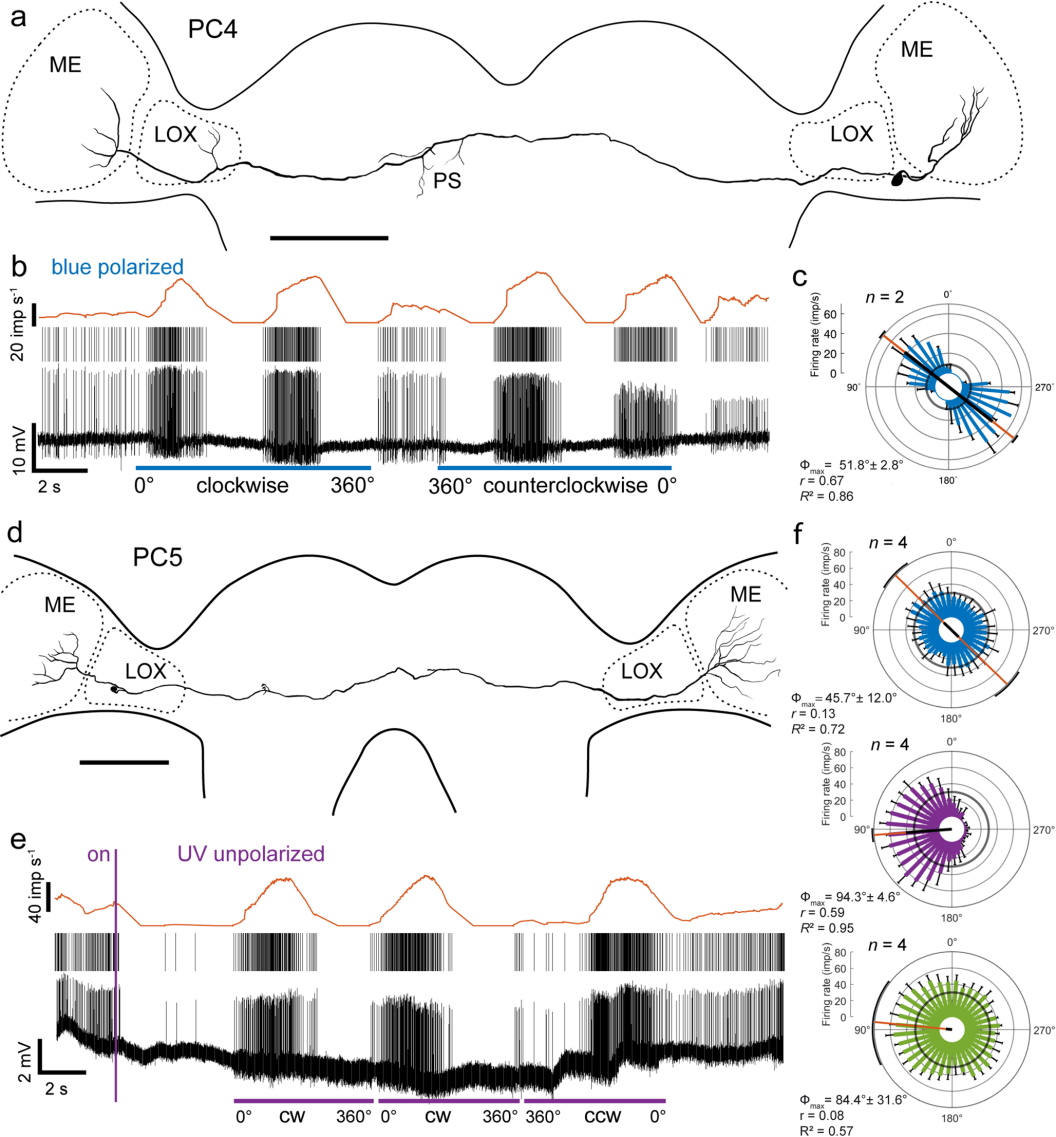
Morphology and physiology of commissural neurons PC4 and PC5. **a** Frontal reconstruction of the recorded PC4 neuron. The neuron connects both medullas (ME) via the posterior optic commissure. Additional ramifications are in the contralateral LOX and the contralateral posterior slope (PS) of the brain. Scale bar = 200 μm. **b** Intracellular recording (bottom trace), raster plot (middle trace), and mean spike frequency (top trace) during a clockwise and counterclockwise rotation of the polarizer (blue horizontal bars; angular velocity 40° s^−1^). Before, between, and after polarizer rotations the animal was exposed to stationary polarized blue light (AoP 0°). **c** Circular plot of spiking activity of the PC4 neuron from one clockwise and one counterclockwise rotation of the polarizer. Spiking activity is plotted in 15° bins (blue bars), black T-bars indicate SD, grey circle indicates background activity. The neuron shows polarization opponency with a preferred AoP at Φ_max_ = 51.8°, illustrated by the orange line with black bar (length of mean vector *r*) and black arcs (95% confidence interval). **d** Frontal reconstruction of the recorded PC5 neuron. The neuron connects both medullas (ME) via the posterior optic commissure. Scale bar = 200 μm. **e** Intracellular recording (bottom trace), raster plot (middle trace), and mean spiking activity (top trace) showing changes in spiking when the unpolarized UV LED is turned on (vertical violet bar, azimuth 0°) and during clockwise (cw) and counterclockwise (ccw) movement of the light spot around the head of the cockroach (angular velocity 60° s^−1^). **f** Circular plots of spiking activity of the PC5 neuron based on 4 stimulations (n) with polarized blue light (top), the unpolarized UV (middle) and green (bottom) light spot. Spiking activity is plotted in 10° bins, black T-bars indicate SD, grey circles indicate background activity. The neuron shows significant responses to the AoP of blue light with Φ_max_ = 45.7°, as well as to the position of the UV (Φ_max_ = 94.3°) and the green light spot (Φ_max_ = 84.4°), as illustrated by the orange lines with black bars (length of mean vector *r*), and black arcs (95% confidence interval).

Two additional commissural neurons with neurites crossing the brain within the posterior optic commissure, termed PC4 and PC5, are characterized here for the first time. Both neurons were labeled only weakly and, therefore, only major processes of both neurons could be traced (Fig. 5a, d). The somata of both neurons were located in the anterior ventral cell cortex between the medulla and LOX. Both neurons had wide tangential ramifications bilaterally in the medulla. In contrast to PC5, PC4 had additional arborizations in the contralateral posterior slope and the contralateral LOX. Both neurons were highly sensitive to visual stimulation. PC4 had a background activity of 9.0 ± 2.1 (SD, *n* = 8) imp s^−1^. During rotation of the polarizer, the neurons showed prominent polarization opponency with a preferred AoP at Φ_max_ = 51.8° (Fig. 5b, c). Stimulation with the unpolarized UV and green LEDs was not tested. PC5 had high background activity of 30.7 ± 4.3 (SD, *n* = 6) imp s^−1^. The neuron was sensitive to the AoP with Φ_max_ = 45.7° (Fig. 5f). The neuron was significantly tuned to the azimuths of the unpolarized light stimuli, especially the UV light. Switching on UV light at 0° resulted in complete inhibition of the neuron (Fig. 5e). The neuron showed spatial opponency to the UV stimulus with Φ_max_ = 94.3° and complete inhibition at angles around 270° (Fig. 5e, f). Tuning to the green LED was at a similar azimuth (Φ_max_ = 84.4°) but there was no inhibition at the opposite azimuth (Fig. 5f).

**Fig. 6:**
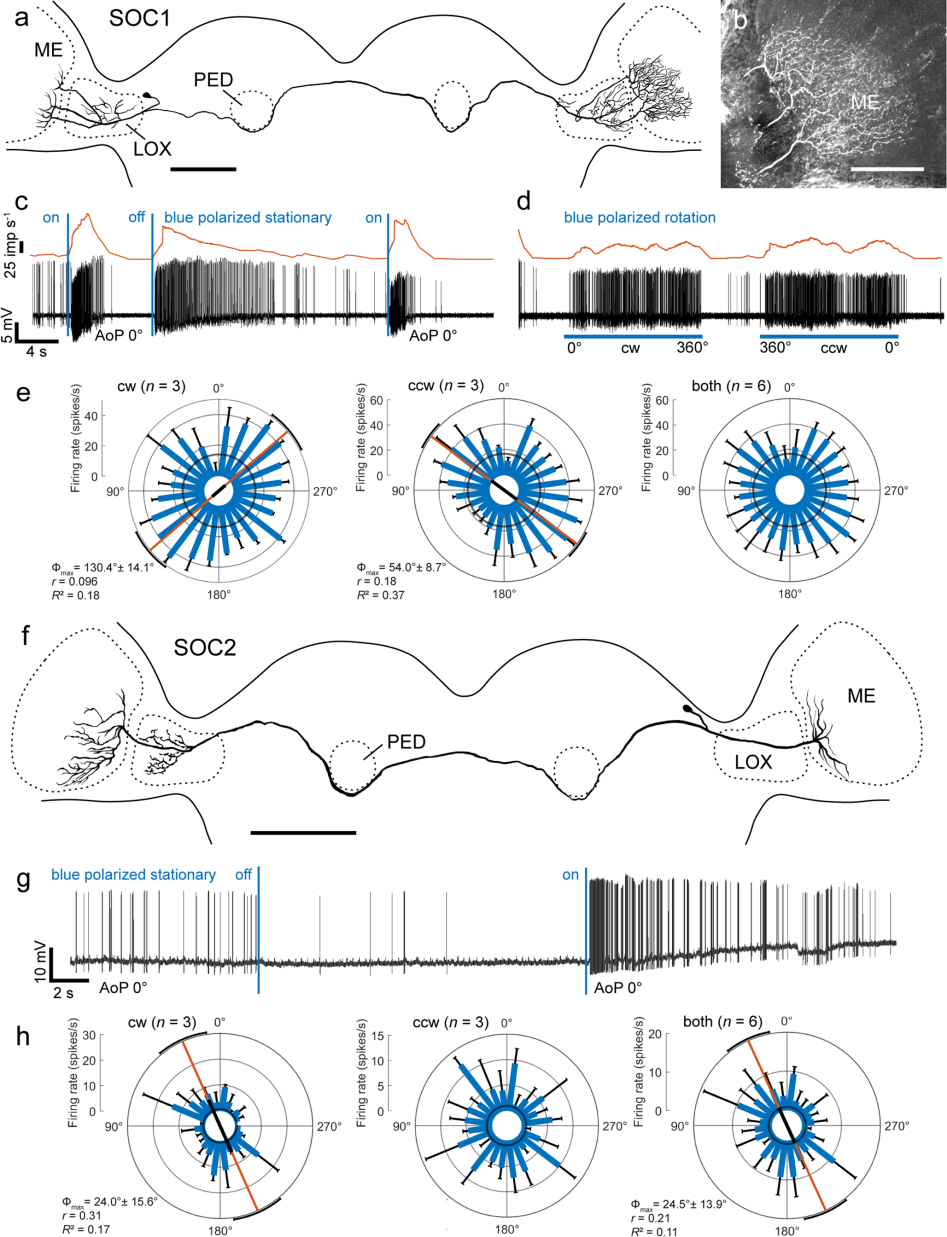
Morphology and physiology of the SOC1 and SOC2 neurons of the serpentine optic commissure. **a** Frontal reconstruction of the SOC1 neuron. It innervates both medullas (ME) and lobula complexes (LOX) but has no ramifications in the central brain. **b** Stack of confocal sections illustrating beaded ramifications in the contralateral ME. **c**,** d** Intracellular recording (bottom) and mean spike frequency (top). **c** The neuron responds to stationary blue polarized light (on/off, blue vertical bars, AoP 0°) with strong phasic excitation after lights on and off followed by complete inhibition. **d** During clockwise (cw) and counterclockwise (ccw) rotation of the polarizer (blue horizontal bars; angular velocity 60° s^−1^) the neuron is highly excited, but shows low spiking activity when the polarizer is kept stationary (before and after the stimuli, AoP 0°). **e** Circular plots of spiking activity during clockwise (cw, left), counterclockwise (ccw, middle), and all polarizer rotations (both, right). Spiking activity is plotted in 15° bins (blue bars); black T-bars indicate SD, grey circles indicate background activity. The neuron shows significant, however nearly orthogonal tuning to AoP during cw and ccw rotations of the polarizer illustrated by the orange lines with black bars (length of mean vector *r*) and black arcs (95% confidence interval). **f** Frontal reconstruction of the SOC2 neuron. It connects both MEs and has additional arborizations in the contralateral LOX. **g** Intracellular recording. The neuron is tonically inhibited when polarized blue light (AoP 0°) is turned off and phasic-tonically excited when light is turned on (blue vertical bars). **h** Circular plots of spiking activity during clockwise (cw, left), counterclockwise (ccw, middle), and all polarizer rotations (both, right; angular velocity 30° s^−1^). Spiking activity is plotted in 15° bins (blue bars); black T-bars indicate SD, grey circles indicate background activity. The neuron shows significant tuning to AoP during cw but not during ccw rotation of the polarizer illustrated by the orange line with black bar (length of mean vector *r*) and black arc (95% confidence interval). Scale bars = 300 μm (a, f), 100 μm (b)

Two recordings were from commissural neurons of the serpentine optic commissure as defined by Reischig and Stengl (2002). The neurons had wide bilateral arborizations in the medulla (Fig. 6). One neuron, SOC1, had, in addition, bilateral ramifications in the outer lobe of the LOX, while the second one, SOC2, innervated only the contralateral LOX (Fig. 6a, b, f). The somata of both neurons were within the cell cortex near the superior medial edge of the LOX, bordering the superior lateral protocerebrum. Axonal fibers passed ventrally around the pedunculi of the mushroom bodies and crossed the brain midline without side branches, in close proximity to the anterior-dorsal face of the central body. SOC1 had a background activity of 14.7 ± 5.0 (SD, *n* = 5) imp s^−1^, SOC2 a low activity of 1.0 ± 1.3 (SD, *n* = 13) imp s^−1^. Both neurons were highly sensitive to blue light stimulation. Green and UV lights were not tested. SOC1 responded with strong phasic excitation to zenithal polarized blue light (AoP 0°) followed by tonic inhibition of spiking (Fig. 6c). A similar but less phasic response occurred when switching the light off (Fig. 6c). The neuron showed high spiking activity during rotation of the polarizer, however with little modulation by polarization angle. Spiking activity completely ceased when rotation of the polarizer stopped, despite continuing illumination of the animal through the stationary polarizer at 0° orientation (Fig. 6d). Although excited by all polarizer orientations, the neuron showed AoP tuning during clockwise and counterclockwise rotation of the polarizer, however with nearly orthogonal Φ_max_ (Fig. 6e). As a consequence, no significant AoP tuning was observed when pooling the responses to clockwise and counterclockwise polarizer rotations (Fig. 6e). The SOC2 neuron, likewise, showed phasic excitation at onset of blue polarized light stimulation (AoP 0°) but, in contrast to SOC1, tonic inhibition when the light was turned off (Fig. 6g). The neuron was, like SOC1, excited by all orientations of the polarizer, it showed tuning to the AoP during clockwise (Φ_max_ = 24.0°) but not to counterclockwise rotation of the polarizer.

### Neurons of the CX

The CX in the cockroach brain consists, like in other insects, of the upper (CBU) and lower (CBL) divisions of the central body, the protocerebral bridge (PB), and a pair of globular-shaped noduli (Althaus et al. 2022; Jahn et al. 2023). We performed intracellular recordings from 25 neurons with arborizations in the CX, including 11 recordings from tangential neurons innervating the PB or distinct layers of the CBU or CBL, 7 recordings from columnar neurons of the CX, and 7 recordings from pontine neurons, connecting columns across the brain midline in the CBU. All neurons were tested for responsiveness to zenithal polarized blue light but only a few neurons for responses to the moving UV and green light spots (Table 1). Overall, only a small fraction of neurons showed angular tuning to the stimuli and even neurons corresponding to cell types of the CX sky compass network in the locust (Homberg et al. 2023) and other insects (Sakura et al. 2008; Heinze and Reppert 2011; el Jundi et al. 2015; Hardcastle et al. 2021; Garner et al. 2024) were responsive only in some recordings and unresponsive in others (Table 1).

### Tangential neurons of the CBL and PB

**Fig. 7:**
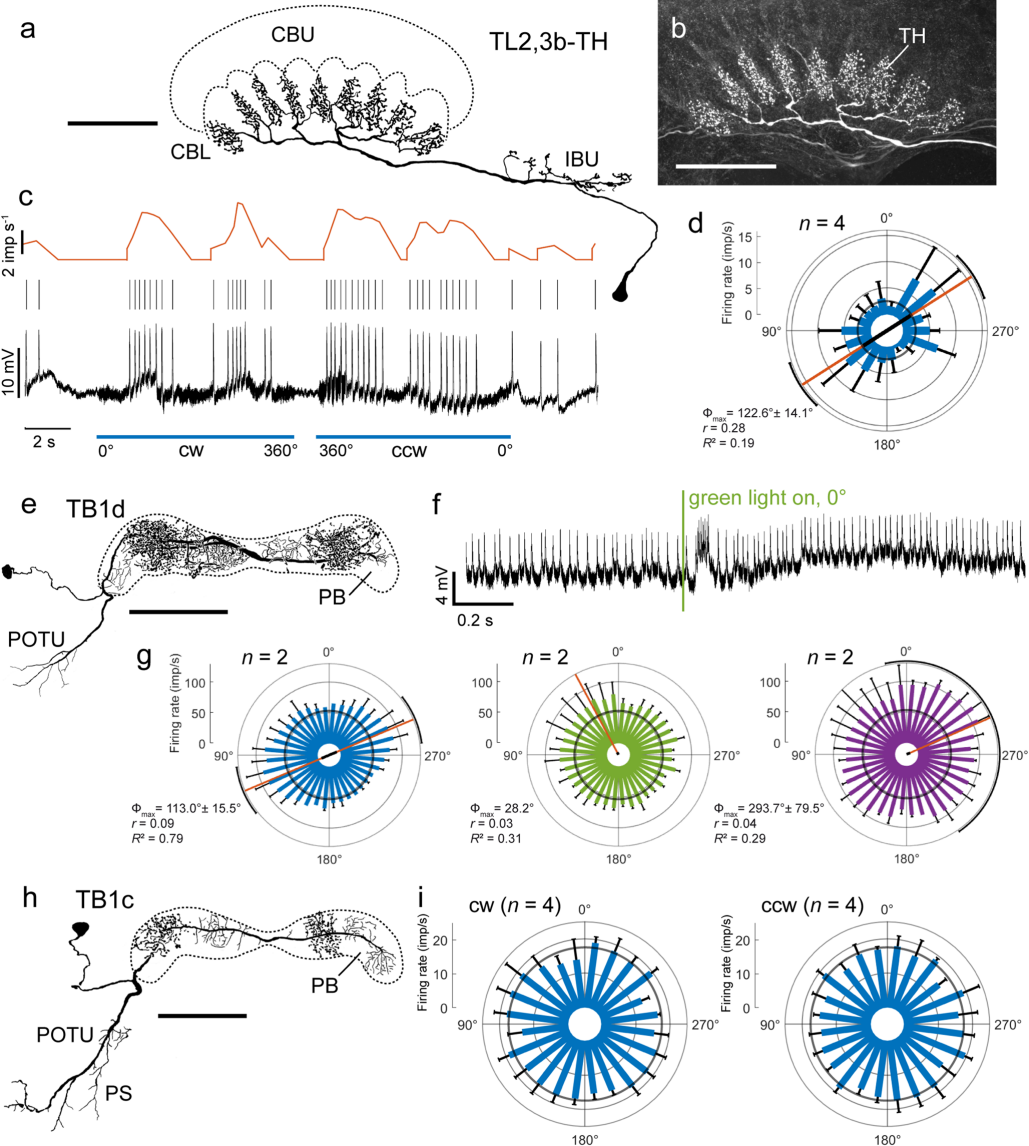
**a–d** Tangential neuron TL2,3b-TH of the lower division of the central body (CBL). **a** Frontal reconstruction of the neuron and **b** confocal image showing ramifications in the central body. The neuron innervates microglomeruli of the inferior bulb (IBU) and sends an axon to beaded arborizations in the center of nine teeth (TH) of the CBL. **c** Spiking activity of the neuron during dorsal illumination of the animal with blue light passing through a polarizer rotating in clockwise (cw) and counterclockwise (ccw) direction (blue bars; angular velocity 40° s^−1^). Bottom trace: intracellular recording, middle trace: raster plot, upper trace: mean spiking activity. **d** Circular plot of spiking activity of the neuron from four rotations (2 clockwise, 2 counterclockwise) of the polarizer. Spiking activity is plotted in 20° bins (blue bars), black T-bars indicate SD, grey circle indicates background activity in the dark. The neuron is maximally excited by an AoP at Φ_max_ = 122.6°, illustrated by the orange line with black bar (length of mean vector *r*) and black arcs (95% confidence interval). **e-g** Tangential neuron TB1d of the protocerebral bridge (PB). **e** Frontal reconstruction of the neuron. The neuron has smooth processes in the posterior optic tubercle (POTU) and two smooth and two varicose ramification domains in the PB. **f** Intracellular recording. The neurons shows a burst of activity following when the green light spot is turned on, followed by a prolonged increase in spiking activity. **g** Circular plots of spiking activity of the TB1d neuron from 2 (*n*) rotations (1 clockwise, 1 counterclockwise) of the polarizer (blue, left), the unpolarized green (middle) and UV (right) light spot (angular velocity 60° s^−1^). Spiking activity is plotted in 10° bins, black T-bars indicate SD, grey circles indicate background activity. The neuron is sensitive to the AoP and the azimuth of the green and UV light spots illustrated by the orange lines with black bar (length of mean vector *r*), and black arcs (95% confidence interval). During stimulation with green and UV light, spiking activity is, in addition, increased independently of stimulus azimuth. **h**,** i** Morphology and physiology of a TB1c neuron of the PB. **h** Frontal reconstruction of the neuron. Outside the PB, the neuron has smooth ramifications in the POTU and in adjacent areas of the posterior slope (PS). **i** Circular plots showing activity during four clockwise (cw) and four counterclockwise (ccw) rotations of the polarizer (angular velocity 30° s^−1^). Spiking activity is plotted in 15° bins (blue bars); black T-bars indicate SD, grey circles indicate background activity. The neuron is insensitive to the AoP. Scale bars = 100 μm

Two recordings were obtained from tangential neurons of the CBL (TL neurons) and three recordings from tangential neurons of the PB (TB neurons). The two TL neurons showed low background spiking activities at 2.5 ± 1.4 (SD, *n* = 5) imp s^−1^ (Fig. 7d) and 4.4 ± 1.1 (SD, *n* = 8) imp s^−1^ (not shown). Only one neuron, a TL2,3b-TH neuron, was sensitive to polarization angle (Fig. 7a–d). The neuron innervated microglomeruli in the lower bulb and invaded the central parts of the nine teeth in the CBL (Fig. 7a, b). Spiking activity was modulated during rotation of the polarizer with a preferred AoP at Φ_max_ = 122.6° based on two clockwise and two counterclockwise rotations of the polarizer (Fig. 7c, d). Responses to the unpolarized light spots were not tested in either recording.

Sensitivity to AoP was tested in three recordings from TB neurons innervating the PB. A TB1d neuron showed spatial tuning to AoP as well as to the green and UV light spots (Fig. 7). The neuron had smooth ramifications in the posterior optic tubercle of the brain and innervated two domains with smooth and two with varicose processes in the PB (Fig. 7e). The neuron showed short phasic bursts when the light stimuli were switched on, most pronounced upon onset of the green LED (Fig. 7f). Spiking activity was modulated during rotation of the polarizer with a preferred AoP at Φ_max_ = 113.0° based on one clockwise and one counterclockwise rotation of the polarizer. Spiking activity was not reduced at anti-perferred AoP relative to background activity measured shortly before stimulation at 51.0 ± 1.4 (SD, *n* = 4) imp s^−1^ (Fig. 7g). In addition the neuron was sensitive to the azimuth of the green (Φ_max_ = 28.2°) and UV (Φ_max_ = 293.7°) light spots. Throughout the green and UV stimulation, the neuron showed increased firing activity.

Another recording was obtained from a TB1c neuron (Fig. 7h, i). The neuron had a background activity of 17.3 ± 1.4 (SD, *n* = 11) imp s^−1^ and was unresponsive to the AoP during clockwise and counterclockwise rotation of the polarizer (Fig. 7i). The third recording was from a TB4 neuron. It’s morphology is shown in Fig. 2a of Jahn et al. (2024). The neuron had wide smooth processes in the posterior slope and lateral complex and beaded ramifications throughout the PB (Jahn et al. 2024). The neuron had low background spiking activity of 4.9 ± 0.4 (SD, *n* = 7) imp s^−1^ interrupted by short bursts of higher activity at regular intervals of about 1 s (not shown). It was not sensitive to AoP. Responses to unpolarized light spots were not tested.

### Columnar neurons of the CX

**Fig. 8:**
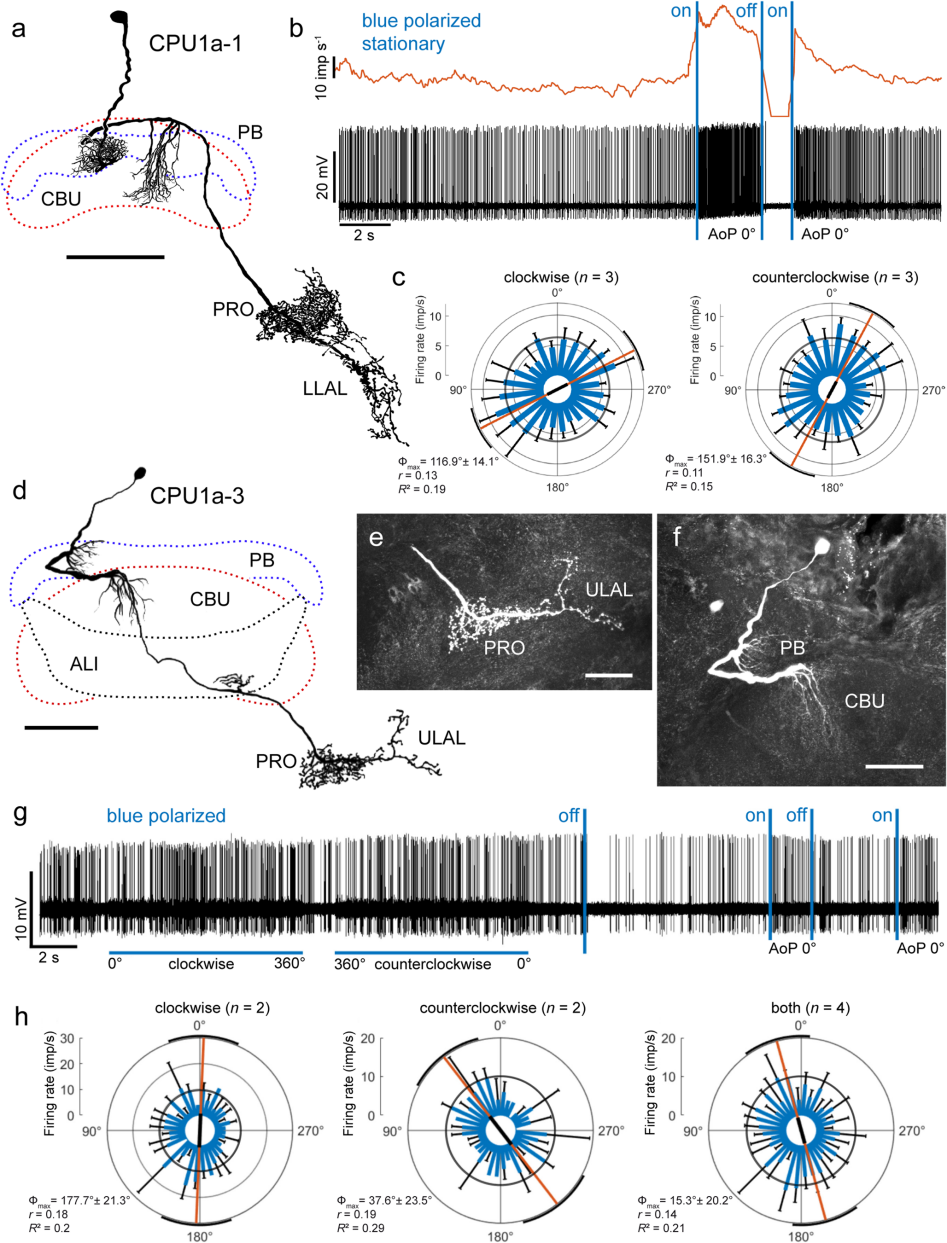
Morphology and physiology of two CPU1a neurons of the central complex. **a** Frontal reconstruction of a CPU1a-1 neuron. The neuron has fine ramifications in a column of the protocerebral bridge (PB) and the upper division of the central body (CBU) and sends an axon via the isthmus tract to beaded terminals in the prong (PRO) and lower lateral accessory lobe (LLAL) of the lateral complex. **b** Intracellular recording (bottom trace) and mean spike frequency illustrating neural activity in the dark. The neuron is highly excited when blue polarized light (blue vertical bars, AoP 0°) is turned on and completely inhibited when the light is turned off. **c** Circular plots of spiking activity during clockwise and counterclockwise rotation of the polarizer (angular velocity 30° s^−1^). Spiking activity is plotted in 15° bins (blue bars); black T-bars indicate SD, grey circles indicate background activity. The neuron shows significant tuning to AoP during both rotations of the polarizer illustrated by the orange lines with black bars (length of mean vector *r*) and black arcs (95% confidence interval). **d** Frontal reconstruction of a CPU1a-3 neuron. The neuron has smooth ramifications in a column of the PB and CBU, additional side branches in the anterior lip (ALI) and beaded terminals in the PRO and upper lateral accessory lobe (ULAL). **e**,** f** Stacks of confocal sections illustrating beaded ramifications in the prong (**e**) and ramifications in the PB and CBU (**f**). **g** Intracellular recording from the CPU1a-3 neuron. Spiking activity is modulated during clockwise and counterclockwise rotation of the polarizer (angular velocity 40° s^−1^). Spiking activity is transiently reduced when polarized blue light (AoP 0°) is turned off, and is enhanced when polarized light is turned on (AoP 0°, blue vertical bars). **h** Circular plots of spiking activity during clockwise (left), counterclockwise (middle), and all polarizer rotations (both, right). Spiking activity is plotted in 10° bins (blue bars); black T-bars indicate SD, grey circles indicate background activity. The neuron shows significant tuning to AoP in all plots illustrated by the orange lines with black bars (length of mean vector *r*) and black arcs (95% confidence interval). Scale bars = 100 μm (a, d), 50 μm (e, f)

Seven recordings were obtained from columnar neurons of the CBU, including two recordings from CU neurons, connecting the CBU to the lateral complex, one recording from a CP neuron, projecting from the PB to the lateral complex, three recordings from CPU1 neurons that innervated the PB, the CBU and the lateral complex, and one recording form a CPU5 neuron, connecting the PB and CBU to the noduli. With few exceptions these neurons were tested only for responsiveness to polarization angle. Of these seven neurons, only two CPU1 neurons were polarization sensitive (Fig. 8). These two neurons, a CPU1a-1 neuron (Fig. 8a-c) and a CPU1a-3 neuron (Fig. 8d-h), showed highly similar responses to polarized light. The neurons had smooth arborizations in single columns of the PB and innervated a column of the CBU (Fig. 8a, d, f). Ramifications in the CBU were concentrated in layer II (Jahn et al. 2023). Axonal fibers gave rise to prominently beaded ramifications in the contralateral prong and posterior areas of the lateral accessory lobe (Fig. 8a, d, e). In contrast to the CPU1a-1 neuron, the CPU1a-3 neuron had additional varicose side branches in the anterior lip (ALI; Fig. 8d). Both neurons had initially high activity (CPU1a-1: 14.5 ± 2.6 (SD, *n* = 8) imp s^−1^, Fig. 8b; CPU1a-3: 18.9 ± 3.8 (SD, *n* = 8) imp s^−1^, Fig. 8g) which later decreased (CPU1a-1: 7.0 ± 1.3 (SD, *n* = 10) imp s^−1^, Fig. 8c; CPU1a-3: 10.1 ± 4.1 (SD, *n* = 8) imp s^−1^, Fig. 8h). Both neurons were sensitive to the angle of polarized light (Fig. 8b, c, g, h). Spiking activity markedly increased when polarized light (AoP 0°) was turned on (Fig. 8b, g). When polarized light (AoP 0°) was turned off again, spiking activity ceased completely (Fig. 8b) or was transiently strongly reduced (Fig. 8g). Like most other neurons both neurons showed some rotation direction-dependent mismatch in tuning to the AoP. The AoP eliciting maximal excitation, determined from three clockwise and counterclockwise rotations was at Φ_max_ = 116.9° (clockwise) and Φ_max_ = 151.9° (counterclockwise) for the CPU1a-1 neuron (Fig. 8c) and, determined from two rotations each, at Φ_max_ = 177.7° (clockwise) and Φ_max_ = 37.6° (counterclockwise) for the CPU1a1-3 neuron (Fig. 8h). Azimuthal tuning to unpolarized green and UV light spots were not tested in either neuron.

### Tangential and pontine neurons of the CBU

**Fig. 9:**
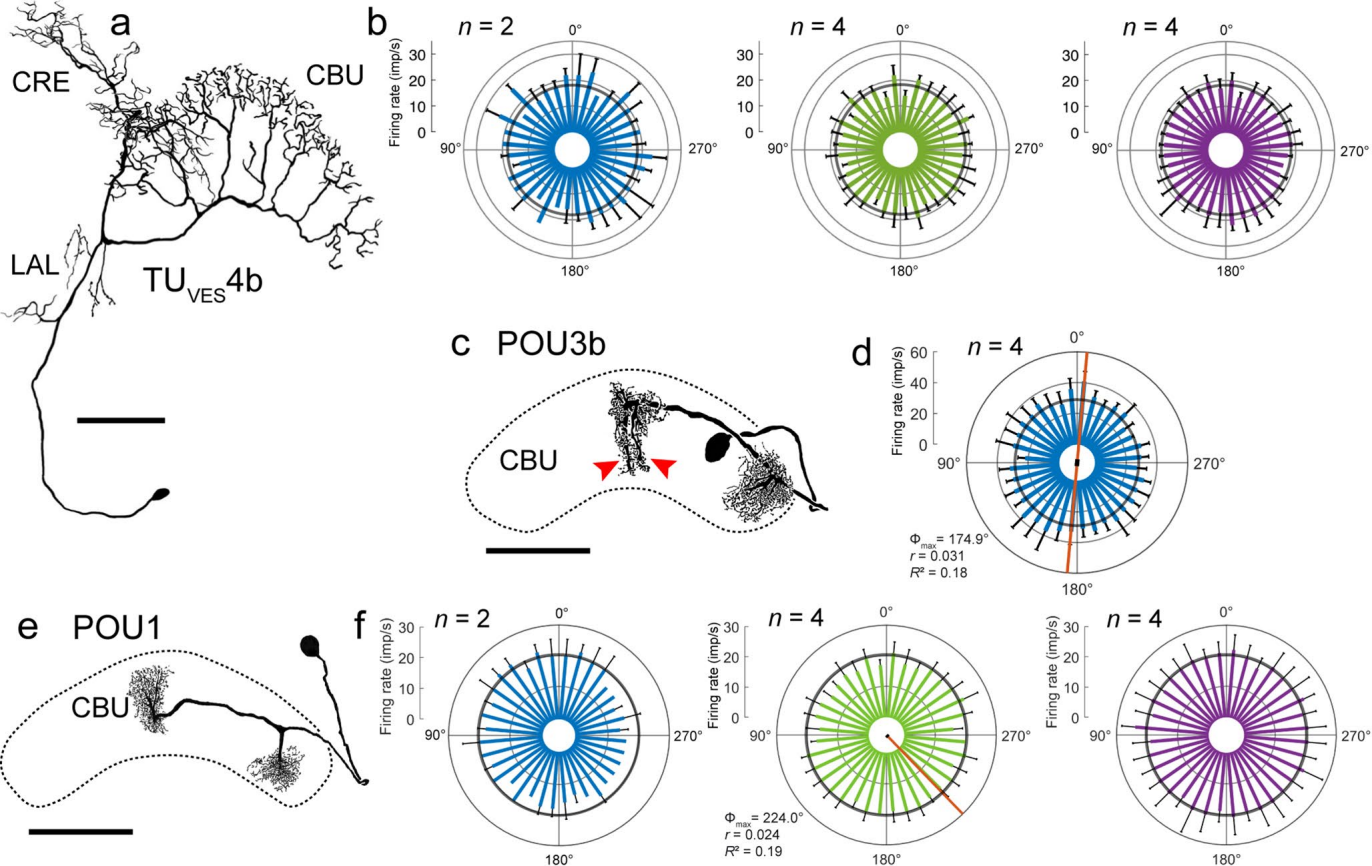
Tangential and pontine neurons of the central complex. **a**,** b** TU_VES_4b neuron. **a** Morphology of the neuron, reproduced from Jahn et al. (2024). The neuron has ramifications in the crepine (CRE) and lateral accessory lobe (LAL) of the left brain hemisphere and varicose processes in a particular layer of the upper division of the central body (CBU). **b** Circular plots showing activity during two rotations of the polarizer (left), and four rotations of the green (middle) and the UV LED (right; angular velocity 40° s^−1^). Spiking activity is plotted in 10° bins (colored bars); black T-bars indicate SD, grey circles indicate background activity. The neuron is insensitive to the AoP and the azimuth of the unpolarized light spots. **c**,** d** POU3b neuron. **c** The neuron has finely beaded ramifications in the right outermost column of the upper division of the central body (CBU) and two parallel trees of varicose ramifications (red arrowheads) in the innermost column of the left hemisphere. **d** Circular plot illustrating activity during two clockwise and two counterclockwise rotations of the polarizer (angular velocity 30° s^−1^). Spiking activity is plotted in 10° bins (blue bars); black T-bars indicate SD, grey circles indicate background activity in the dark. The neuron shows preference for an AoP at 174.9°, illustrated by the orange line with black bar (length of mean vector *r*). **e**,** f** POU1 neuron. **e** The neuron has smooth ramifications in the right hemisphere of the CBU and finely beaded processes in a column of the left CBU hemisphere. **f** Circular plots showing mean activity during one clockwise and one counterclockwise rotation of the polarizer (left) and two clockwise and two counterclockwise rotations of the green (middle) and UV LED (right; angular velocity 40° s^−1^). Spiking activity is plotted in 10° bins (colored bars); black T-bars indicate SD, grey circles indicate background activity. The neuron is insensitive to the AoP and azimuth of the UV light, but shows preference for the green LED at Φ_max_ = 224.0° illustrated by the orange line with black bar (length of mean vector *r*). Scale bars = 100 μm

Responsiveness to sky compass signals was tested in six recordings from TU neurons, comprising five TU_VES_ neurons and one TU_PS_ neuron. None of these neurons showed a preferred AoP during rotation of the polarizer or a preferred azimuth when testing the unpolarized light spots. An example is shown in Fig. 9a, b. The neuron had a background activity of 18.0 ± 3.6 (SD, *n* = 10) imp s^−1^.

Seven recordings were obtained from pontine neurons of the CBU. One of these neurons, a novel POU3b neuron, showed weak preference for the AoP (Fig. 9c, d). In contrast to previously described POU3 neurons characterized by two parallel arms of their proximal dendritic tree in a column of the CBU (Jahn et al. 2023), the POU3b neuron had two parallel arms in its distal axonal tree (red arrowheads in Fig. 9c). The neuron showed background spiking activity of 28.6 ± 2.1 (SD, *n* = 5) imp s^−1^ and preference for an AoP at 174.9° (Fig. 9d). Another neuron, characterized as POU1 (Fig. 9e) showed weak preference for the azimuth of the green light spot at Φ_max_ = 224.0° but no tuning preference to polarized light or the UV light spot (Fig. 9f). Its background spiking activity was 20.8 ± 3.4 (SD, *n* = 9) imp s^−1^.

## Discussion

We show here that neurons of the optic lobe and CX in the brain of the Madeira cockroach *R. maderae* are sensitive to celestial cues, in particular the AoP of light from dorsal direction suggesting that these cockroaches, like many other insect species, rely on sun compass cues for orientation in space. Our study confirms and extends previous accounts on polarization-sensitive neurons in the optic lobe of the cockroach *Periplaneta americana* (Kelly and Mote 1990) and *R. maderae* (Loesel and Homberg 2001) and is the first to provide evidence for the processing of sky compass signals in neurons of the cockroach CX, a brain area which plays a pivotal role in spatial orientation and navigation (Varga et al. 2017; Honkanen et al. 2019; Heinze 2023).

### Neurons of the optic lobe

Six commissural neurons connecting both optic lobes (Figs. 4, 5 and 6) and a projection neuron from the LOX to the central brain (Fig. 2) were sensitive to the AoP of zenithal light. In addition, one commissural neuron (Fig. 5d–f) and an ocellar interneuron projecting to the LOX and medulla (Fig. 3) were tuned to the direction of the unpolarized light spots (Table 1). The recorded ocellar interneuron OC2 most likely corresponds to ocellar interneuron OL1 in the cockroach *Periplaneta americana* (Mizunami and Tateda 1986; Mizunami 1995a). Six ocellar interneurons, termed OL1-6, have been identified in the brain of the American cockroach, projecting from the ocellar nerve to the optic lobe (Mizunami 1995a). While no recordings exist of OL5 and 6, spiking in OL1-4 is inhibited by ocellar illumination (Mizunami and Tateda 1986; Mizunami 1995b). The OC2 neuron, recorded here in the Madeira cockroach, was, likewise, inhibited by light spots moving around the head of the animal, but showed lack of inhibition and slight excitation at an orientation of the stimulus around 180°, i.e. behind the animal, both for the UV and the green light. As the two ocelli, positioned between the rim of the compound eyes and the sockets of the antennae, are directed frontally, the LED stimuli at an elevation of 45° and azimuth around 180° may be beyond the receptive fields of the ocelli, which might explain the apparent azimuth-dependent responses. The sensitivity to green and UV light suggests that, in contrast to the American cockroach which possesses only green-sensitive ocellar photoreceptors (Goldsmith and Ruck 1958), the ocelli of the Madeira cockroach might contain both UV- and green-sensitive photoreceptors.

Polarization sensitivity in two commissural neurons, PC2 and PC3, has been demonstrated by Loesel and Homberg (2001) and is confirmed here. The preferred AoP in PC2 neurons were at 35.6° (this study), and 38° and 85° in two recordings from Loesel and Homberg (2001). Preferred polarization angles for the PC3 neuron were at 72° and 77° in two recordings from Loesel and Homberg (2001) and at 136.4° in this study. It is, therefore likely, that PC2 and PC3 consist of several individual neurons with identical morphology but different tuning angles to polarized light, as has been shown for commissural POL1 neurons of crickets, which occur as three bilateral pairs with preferred AoP differing by about 60° (Labhart and Petzold 1993; Labhart and Meyer 2002). PC2, like the highly similar polarization-sensitive POL1 neurons in the cricket (Labhart and Petzold 1993) and the MeMe1 neuron in the locust (el Jundi et al. 2011) innervate the accessory medulla, the master circadian clock in the cockroach brain (Stengl et al. 2015). Whether this connection plays a role in time compensation of sun compass orientation or, alternatively, contributes to entrainment of the clock, remains to be studied.

Two additional neurons connecting the two medullas via the posterior optic commissure, termed PC4 and PC5, were highly sensitive to polarization angle illustrated by *R*^2^ values > 0.7. One of these neurons, PC4, sent side branches to the posterior slope, in close proximity to dendritic ramifications of TB neurons (Fig. 7e; Jahn et al. 2023) and might therefore, provide polarization signals to the CX. Circular tuning of the second neuron, PC5, to the unpolarized light spots suggests, in addition, tuning by direct input from sunlight.

Two recordings from commissural neurons connecting both optic lobes via the serpentine optic commissure, SOC1 and SOC2, showed activity changes during presentation of polarized light that were strikingly different from the compass-like responses of the commissural neurons in the posterior optic tract. The cause of the orthogonal Φ_max_ orientations of the SOC1 neuron to clockwise and counterclockwise rotations is at present difficult to assess. The neuron exhibited complex and highly unusual phasic dynamics in its response to polarized light: when polarized light was turned on or off, it responded with a burst of activity followed by complete inhibition. During changes in polarization angle, the excitatory component apparently generated by the changing AoP appears to strongly override the inhibitory aftereffects, which, however, may eventually influence the response by slightly modulating the strong excitation during polarizer rotation. If the temporal dynamics of this interaction are similar for clockwise and counterclockwise rotations, a decrease of activity after approximately the same time following onset of rotation would occur resulting in turn-direction dependent orthogonal Φ_max_ orientations. The tuning of the SOC2 neuron to polarization angle when rotating the polarizer in one direction but not the other suggests that this neuron plays a role in distinguishing the direction of yaw rotation through turn-direction dependent changes in the perceived sky polarization pattern.

Morphologically similar neurons connecting the medulla and LOX of both optic lobes via the serpentine commissure have been studied in bees (Hertel et al. 1987; Honkanen et al. 2023). Like the SOC neurons of the cockroach, those neurons showed phasic responses to lights on and off followed by tonic inhibition. The neurons were highly sensitive to flow field motion in the yaw plane with low or no direction sensitivity. Sensitivity to polarized light was not tested. The response characteristics of these neurons in bees and the cockroach might nicely complement each other. In both species the neurons are highly excited during yaw-turns of the insect, perceived through large-field motion of the environment and/or rotation below the sky. It will be interesting to study whether and how terrestrial and celestial inputs are integrated in these neurons to uncover their role in controlling locomotion.

The LOX projection neuron, LOX-PN1, is a promising candidate to provide polarization and thus sky compass input to descending pathways. The neuron receives input in the LOX and has wide bilateral projections to the posterior slope, inferior bridge, and vest, areas that are prominently innervated by dendritic processes of descending neurons (Okada et al. 2003). The mismatch in the AoP eliciting maximal excitation depending on rotation direction of the polarizer, found in LOX-PN1 (Fig. 2d) and other neurons (e.g. Figure 8c, h), is a wide-spread phenomenon of polarization-sensitive neurons, previously reported in locusts (Bockhorst and Homberg 2015; Beetz et al. 2016), crickets (Sakura et al. 2008) and bumblebees (Rother et al. 2024). It is characterized by shift of Φ_max_ against the direction of rotation during low rotation velocities and may serve to anticipate future head directions as suggested for certain head-direction cells in rats (Blair and Sharp 1995).

### Neurons of the CX

 Based on evidence derived from extracellular recordings for a major role of the cockroach CX in spatial visual orientation and motor control (Ritzmann et al. 2012; Varga et al. 2017) we aimed at characterizing the responsiveness of CX neurons of the Madeira cockroach brain to sky compass cues, including polarization angle and chromatic cues. Surprisingly most CX neurons were relatively insensitive to these stimuli (*R*^2^ < 0.3; Figs. 7, 8, 9; Table 1), despite an anatomical organization of the CX highly similar to that in other insect species (Althaus et al. 2022; Jahn et al. 2023, 2024). Several reasons might contribute to the weak or completely absent responses. The spectral sensitivity of photoreceptors that provide polarized-light signals to the CX is not known and might not align with the blue light used for testing polarization sensitivity here. A previous study on the PC2 neuron of the optic lobe showed that polarization sensitivity was abolished by inserting a long pass filter (cutoff wavelength 471 nm) into the light beam generated by a xenon arc lamp (Loesel and Homberg 2001). This suggests that polarization-sensitive photoreceptors of the eye are either UV- or blue-sensitive. If these photoreceptors are UV-sensitive, as is the case in bees, ants, and flies, the blue light used here may have only partially stimulated them, thereby leading to weak responses in CX neurons. Being a nocturnal insect, celestial bodies like the sun or the daytime polarization pattern of the sky may, in addition, be less important for spatial orientation than in diurnal species. Another nocturnal insect, the Bogong moth, relies on the Earth’s magnetic field and a stellar compass for spatial orientation (Dreyer et al. 2018, 2025). Intracellular recordings from various cell types, including CX neurons, showed tuning to the orientation of a starry sky (Dreyer et al. 2025). Therefore, nighttime cues, such as a starry sky, might also be more effective in eliciting spatial tuning responses in the CX neurons of the cockroach. Finally, as a scavenger, the Madeira cockroach may place less importance on targeting specific items than its close relative, the praying mantis which uses visual cues to locate prey targets by angle and distance. Wosnitza et al. (2022) showed that CX neurons in the mantis are prominently involved in these tasks which may be of little relevance to the Madeira cockroach. Comparing the spatial tuning properties of CX neurons in the two dictyopteran species showing strikingly different behaviors may thus be a highly interesting area of research in the future.

Studies on various species showed that the pathway for sky compass signals to the CX is highly conserved among insects. It involves photoreceptors in dorsal rim areas and other parts of the eyes, interneurons passing signals through the optic lobe, the lower units of the anterior optic tubercle, and the bulbs of the lateral complex to the CX (Homberg et al. 2011; Honkanen et al. 2019). The existence of this pathway has not been fully established in cockroaches. Evidence for the presence of a polarization-sensitive dorsal rim area in the Madeira cockroach is lacking and is mixed for other cockroach species (Labhart and Meyer 1999). In contrast to many other insect species, however, high polarization sensitivity (PS ~ 5) has been reported for photoreceptors throughout the eye of the American cockroach *Periplaneta americana* (Butler and Horridge 1973). The lower unit of the anterior optic tubercle, intimately involved in sky compass signaling in all insects studied so far, is small in *R. maderae* (Althaus et al. 2022). Nevertheless, neurons of the tubercle-bulb tract connecting the lower unit of the tubercle to the bulb, are present, and likely contact TL input neurons to the CX (Althaus et al. 2022).

Within the CX a core network of neurons processing sky compass signals, including subtypes of TL neurons (ER neurons in flies), CL neurons (EPG neurons in flies), TB neurons (Δ7 neurons in flies), and CPU neurons (PFL neurons in flies) has been identified in several species, notably locusts (Pegel et al. 2018; Homberg et al. 2023), butterflies (Heinze and Reppert 2011), beetles (el Jundi et al. 2015), bees (Stone et al. 2017; Rother et al. 2024), and fruit flies (Hardcastle et al. 2021). Five neurons of the cockroach CX, a TL neuron, two CPU1 neurons, a TB neuron, and a POU neuron were sensitive to zenithal AoP and, thus, might play a role in sun compass orientation. In flies, most ER/TL neurons provide visual input to the CX but only a fraction is sensitive to the AoP (Hardcastle et al. 2021; Garner et al. 2024). Other ER/TL neurons signal direct sunlight, other visual cues, or information on wind direction. This might explain why only one of two TL neurons in the cockroach was responsive to AoP. CPU1 neurons are the major sky compass output elements of the CX. In the cockroach, two of three CPU1 neurons were sensitive to zenithal polarized light suggesting that their ensemble might constitute an internal sky compass, as shown in locusts and other species.

Several other cell types of the cockroach CX, notably most POU neurons (hΔ neurons in flies), all CU neurons (FX neurons in flies), and TU neurons (FB neurons in flies), were insensitive to sky compass signals, which corresponds to lack of evidence for direct involvement in sky compass signaling in all other species studied. Weir and Dickinson (2015) showed in flies that sensory signal processing in the CX is highly affected by motor activity of the insect and may be strongly suppressed during immobility. This may at least in part explain the apparent lack of responsiveness to our stimuli in many CX neurons.

### Biological significance

The data show that, in addition to light intensity and wavelength, the third quality of light, the polarization angle, is prominently represented and analyzed in the cockroach brain. The most important source of polarized light is skylight, generated by scattering of sunlight in the atmosphere. Therefore, sensitivity to polarization angle contributes to internal control of goal orientation in space. Other sources of polarized light are reflections from shiny surfaces such as bodies of water, vegetation, or, as shown in parasitic horseflies, dark animals allowing for host detection (Horváth 2024).

Cockroaches are excellent navigators. Although direct evidence from *R. maderae* is lacking, other cockroach species show sophisticated orientation skills during climbing (Watson et al. 2002), multisensory spatial learning (Kwon et al. 2004; Pomaville and Lent 2018), chemotaxis (Bell and Tobin 1981; Willis and Avondet 2005), and escape (Bell et al. 1983). German cockroaches, *Blattella germanica*, efficiently find back to a shelter and are able to perform path integration even in complete darkness (Durier and Rivault 1999; Rivault and Durier 2004). Electrical stimulation experiments showed that the CX controls movement direction in the discoid cockroach *Blaberus discoidalis* (Martin et al. 2015), based in part by encoding of orientation relative to visual landmarks (Varga et al. 2017). Evidence provided here suggests that, in addition to panorama information, celestial cues such as sky polarization contribute to the representation of space in the CX like in other insect species. Future studies might aim to uncover the impact of these signals during orientation in freely walking cockroaches.

## Data Availability

All data that support the findings of this study are available from the corresponding author.

## Abbreviations

AoP: Angle of polarization
CBL: Lower division of the central body
CBU: Upper division of the central body
CX: Central complex
SD: Standard deviation
LOX: Lobula complex
PB: Protocerebral bridge
